# Trained Immunity in *Anopheles gambiae*: Antibacterial Immunity Is Enhanced by Priming via Sugar Meal Supplemented With a Single Gut Symbiotic Bacterial Strain

**DOI:** 10.3389/fmicb.2021.649213

**Published:** 2021-04-30

**Authors:** Aditi Kulkarni, Ashmita Pandey, Patrick Trainor, Samantha Carlisle, Jainder S. Chhilar, Wanqin Yu, Alex Moon, Jiannong Xu

**Affiliations:** ^1^Department of Biology, New Mexico State University, Las Cruces, NM, United States; ^2^Department of Economics, Applied Statistics and International Business, New Mexico State University, Las Cruces, NM, United States; ^3^Department of Chemistry and Biochemistry, New Mexico State University, Las Cruces, NM, United States

**Keywords:** mosquito, priming, trained immunity, RNA-seq, *Anopheles gambiae*, transcriptomic response

## Abstract

Mosquitoes have evolved an effective innate immune system. The mosquito gut accommodates various microbes, which play a crucial role in shaping the mosquito immune system during evolution. The resident bacteria in the gut microbiota play an essential role in priming basal immunity. In this study, we show that antibacterial immunity in *Anopheles gambiae* can be enhanced by priming via a sugar meal supplemented with bacteria. *Serratia fonticola* S1 and *Enterobacter* sp. Ag1 are gut bacteria in mosquitoes. The intrathoracic injection of the two bacteria can result in an acute hemocoelic infection in the naïve mosquitoes with mortality of ∼40% at 24 h post-infection. However, the *Enterobacter* or*Serratia* primed mosquitoes showed a better 24 h survival upon the bacterial challenge. The priming confers the protection with a certain degree of specificity, the *Enterobacter* primed mosquitoes had a better survival upon the *Enterobacter* but not *Serratia* challenge, and the *Serratia* primed mosquitoes had a better survival upon the *Serratia* but not *Enterobacter* challenge. To understand the priming-mediated immune enhancement, the transcriptomes were characterized in the mosquitoes of priming as well as priming plus challenges. The RNA-seq was conducted to profile 10 transcriptomes including three samples of priming conditions (native microbiota, *Serratia* priming, and *Enterobacter* priming), six samples of priming plus challenges with the two bacteria, and one sample of injury control. The three priming regimes resulted in distinctive transcriptomic profiles with about 60% of genes affected by both bacteria. Upon challenges, different primed mosquitoes displayed different transcriptomic patterns in response to different bacteria. When a primed cohort was challenged with a heterogenous bacterium, more responsive genes were observed than when challenged with a homogenous bacterium. As expected, many canonical immune genes were responsive to the priming and challenge, but much more non-immune genes with various functions were also responsive in the contexts, which implies that the prior priming triggers a delicately coordinated systemic regulation that results in an enhanced immunity against the subsequent challenge. Besides the participation of typical immune pathways, the transcriptome data suggest the involvement of lysosome and metabolism in the context. Overall, this study demonstrated a trained immunity via priming with bacteria in diet.

## Introduction

Invertebrate organisms have evolved an effective innate immune system throughout evolution. The immune mechanisms effectively counteract various infectious agents such as viruses, bacteria, fungi, and parasites. The innate immune machinery is genetically encoded, consisting of pattern recognition receptors, immune pathways, and immune effectors. Upon recognition of pattern molecules from each type of invaders, respective immune pathways will be activated to produce relevant effectors to the pathogens (Baxter et al., 2017; Shaw et al., 2018). Although the innate immunity in invertebrates lacks the immune memory and specificity in a form defined as in the adaptive immunity in vertebrates, increasing evidence indicates that in invertebrates the immune efficacy can be enhanced by immune priming. In such cases, prior exposure to a pathogen can trigger better protection against a repeated challenge, i.e., “priming” followed by “challenge.” Such phenomena have been defined as innate immune memory or trained immunity (Milutinovic and Kurtz, 2016; Netea and van der Meer, 2017; Gourbal et al., 2018; Melillo et al., 2018; Netea et al., 2020; Sharrock and Sun, 2020). In mosquitoes, priming effects have been demonstrated in several contexts. The presence of midgut microbiota is essential to prime and maintain a basal immunity against malaria parasite *Plasmodium falciparum* (Dong et al., 2009). There is a crosslinked physical barrier between gut microbes and gut epithelial cells, which limits the microbial immune elicitors to be sensed by epithelial cells and allows the immune permeability to the gut microbiota (Kumar et al., 2010). The *P. falciparum* invasions breach this barrier and trigger the immune response against bacteria, in turn, this heightened immunity indirectly enhances the resistance against *P. falciparum* (Rodrigues et al., 2010). *Asaia* is a gut bacterium present predominantly in the gut microbiota. The introduction of *Asaia* into midgut via diet modulated the transcription of certain immune genes in *Anopheles stephensi* and *Anopheles gambiae*, and this microbial manipulation elevated anti-*Plasmodium* immunity in *Anopheles stephensi* but not in *An. gambiae* (Cappelli et al., 2019). In addition, one of the two strains of *Serratia marcescens*, which were isolated from wild caught specimens of *Anopheles sinensis*, was able to inhibit the development of rodent malarial parasite *Plasmodium berghei* in *An. stephensi* (Bai et al., 2019). The colonization of the *Serratia* strain in the gut primed the antimalaria effect via modulating the immune genes including antimalaria effectors (Bai et al., 2019). The interactions with bacteria in the microbiota play a critical role in shaping mosquito immune system throughout the evolution. Priming effects have also been investigated in the immunity against bacteria. Brown et al. (2019) have shown that the treatment of *An. gambiae* larvae by injecting *Escherichia coli, Enterobacter* sp., or *Staphylococcus aureus* increased the number of circulating hemocytes and enhanced phagocytosis upon a challenge with *E. coli* in the eclosed adults. However, the prior infection with *E. coli* in larvae did not affect the survival upon the *E. coli* challenge in adults (Brown et al., 2019). In *An. gambiae* adults, a prior hemocoelic infection by injecting *E. coli* primed a stronger immunity against a second *E. coli* infection. The primed mosquitoes had more circulating hemocytes and elevated expression of *NOS* and *PPO6* genes upon the secondary infection (Powers et al., 2020). To further understand the innate immune system in mosquitoes, we examined the priming effect of gut bacteria on immune response in this study. *An. gambiae* mosquitoes were primed in sugar diet with native microbiota, and sugar meal supplemented with bacteria *Enterobacter* or *Serratia*, respectively. Post priming, we examined the effect on survival upon the challenge with the homogeneous or heterogeneous bacteria, respectively. Further, we analyzed the systemic transcriptomic response to the priming as well as the challenges using a homogeneous or heterogeneous bacterium in the primed mosquitoes.

## Materials and Methods

### Mosquitoes

*Anopheles gambiae* Giles sensu stricto G3 strain was obtained from MR4 and was reared at 28°C with 80% humidity under a 10/14 h day–night light cycle. Larvae were fed on a diet of Brewer’s yeast and cat food powder (1:2 ratio). Adults were maintained on 10% sucrose daily, and 5-day old females were fed on NIH Swiss outbred mice for blood meal to induce egg production. Eggs were collected on day 3 post blood feeding and placed in water pans.

### Bacterial Feeding

Newly emerged adult mosquitoes excrete meconium (Moll et al., 2001) and initiate a new gut microbiota. Therefore, it is an appropriate time to establish a microbial community by introducing bacteria in the sugar meal. *Enterobacter* sp. Ag1 was originally isolated from the midgut of the G3 strain (Jiang et al., 2012). *Serratia fonticola* S1 was isolated from midgut of the wild-caught specimens of *Aedes albopictus* in Florida in July 2015. The *Enterobacter* strain is persistently present with G3 strain. The *Serratia* strain is not a part of the gut community in the G3 mosquitoes. We used a PCR assay to examine the presence of *Serratia* and *Enterobacter* in the midgut DNA from the G3 mosquitoes. The primer sets targeting two *Serratia* genes, *DNA gyrase* and *glucose phosphatase*, and one *Enterobacter* gene, *DNA gyrase*, were used for PCR. Primer sequences were provided in Supplementary Table 1. The bacteria were tagged with GFP expressing plasmid using a method we described previously (Pei et al., 2015). The 10% sucrose sugar meal was supplemented with respective bacteria at OD_600_ of 1.0. The bacterial sugar meal was given to mosquitoes post eclosion for 3 days, and midgut at day 1 and day 3 post feeding was dissected to examine the presence of GFP tagged bacteria under a fluorescent microscope as described previously (Pei et al., 2015). Both *Enterobacter* and *Serratia* were observed in the gut. Three priming regimes were used, group I was given regular sugar meal without bacterial supplement, defined as native priming; group II was given sugar meal supplemented with *Enterobacter*, defined as *Enterobacter* priming; and group III was given sugar meal supplemented with *Serrati*, defined as *Serratia* priming.

### Bacterial Injection

Bacteria *Enterobacter* and *Serratia* grow overnight in Luria Bertani broth containing ampicillin (100 μg/ml) at 28°C. Bacterial culture was normalized to OD_600__nm_ = 1 and diluted with sterile H_2_O to yielded approximately 1000 colony forming unit (CFU)/μl. On day 4 post the respective priming regimes described above, individual mosquitoes were injected with ∼100 nl of the bacterial solution, and approximately 100 bacterial cells were received per mosquito. Sterile water was injected as injury control. Survival at 24 h post infection was used to assess the antibacterial immunity. On 24 h post injection, mosquitoes were surface cleaned with dipping into two tubes of 70% ethanol sequentially, 15 s each. After cleaning, the thorax of an injected mosquito was homogenized in 50 μl sterile water, and 30 μl homogenates were spread to an LB plate with Ampicillin and cultured at 28°C overnight. The colonies on the plate were examined under UV light to visualize GFP tagged bacteria. In bacteria-injected mosquitoes, GFP-tagged bacteria were recovered, while in sterile water injected mosquitoes no GFP tagged bacteria were detected. The data were generated from three experimental replicates, each replicate had ∼40 females for injection. The survival rates between the cohorts were compared using Chi-square test.

### Transcriptome Analysis

**FIGURE 1:**
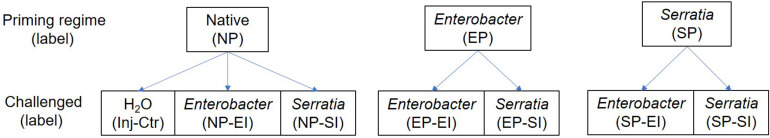
Scheme of priming, challenge, and sampling for RNA-seq. Newly emerged *An. gambiae* G3 mosquitoes were set into three cohorts for priming treatment via sugar meal: native microbiota, supplemented with *Enterobacter* and *Serratia*. The primed cohorts were then subject to *Enterobacter* and *Serratia* challenge, respectively. The sterile H_2_O injection in native primed cohort was used as injury control. RNA samples were extracted from each cohort for RNA-seq, a total of 30 samples were collected from 10 conditions with three replicates for each condition.

RNA-sequencing (RNA-seq) was used to compare transcriptomes. The samples of a total of 10 conditions were collected for RNA-seq, which included three priming regimes, each priming regime had two challenges with homogeneous and heterogeneous bacteria, and sterile water injection in mosquitoes with native community was used as injury control. Each condition had three replicates, therefore 30 samples were collected for RNA-seq. The scheme of bacterial priming, challenge, and RNA sampling was presented in Figure 1. The SRA biosample ID was listed in Supplementary Table 6. For each sample, RNA was isolated from 20 mosquitoes. The whole mosquitoes were used for RNA extraction using Trizol reagent (Invitrogen), and TURBO DNase I (Invitrogen) treatment was followed to remove genomic DNA contamination. The RNA samples were shipped to Genewiz for further processing to make cDNA libraries for sequencing using Illumina Hiseq, 2 × 150 bp paired-end chemistry. At least 25M clean reads were generated from each RNA sample, which provided a sequencing depth sufficient for transcriptome analysis. The reads were mapped against *An. gambiae* reference of transcripts (NCBI), which was implemented by using Array Star v.16 (DNAstar). Read counts were normalized using the median of ratios method (Li et al., 2020) using DESeq2 software (Love et al., 2014). In determining normalized read counts, this method accounts for sequencing depth and RNA composition by calculating normalization factors for each sample in comparison to a pseudo-reference sample. After determining normalized read counts, an independent filter was utilized which removed transcripts with normalized counts less than 5. This resulted in a dataset of 10,689 transcripts (Supplementary Table 2). The clustering of all samples revealed that replicate 2 of *Enterobacter* priming-*Serratia* infection was not consistent with the other two replicates, likely due to a quality issue, therefore, this replicate was removed from the analysis. Differentially expressed genes were identified using a negative binomial generalized linear model (GLM) available through DESeq2 (Love et al., 2014). Likelihood ratio tests were conducted to identify transcripts that exhibited differential expression between all groups. Pairwise differential expression comparisons were made, and statistical significance was determined by computing *q*-values that preserve the False Discovery Rate (FDR) (Storey, 2003; Storey and Tibshirani, 2003; Li et al., 2020). For example, concluding that a transcript was differentially expressed between two groups with a *q*-value of 0.05 would imply that there was a 5% chance (expected) that this conclusion was a false positive. To determine a lower dimensional representation of the transcriptomic data, principal components analysis (PCA) was conducted using regularized log-transformed (rlog) data. PCA seeks to find a small set of “principal components” that capture a large proportion of the variance in the original data (Johnson, 2019). The rlog data was determined using DESeq2, while the “prcomp” function in R (R Core Team, 2019) was utilized to determine the PCA. The proportion of the variance captured by each of the principal components was determined. To validate expression patterns revealed by RNA-seq, a selected set of genes was measured using quantitative RT- PCR. For each sample, RNA was extracted from 15 females using Trizol reagent. Genomic DNA contamination was removed by DNase I treatment as described above. cDNA synthesis was carried out using NEB ProtoScript II Reverse Transcriptase (NEB). The cDNA was used as a template for RT-PCR to determine the transcript abundance of target genes. Three cohorts of primed mosquitoes were challenged with homogeneous and heterogenous bacteria, respectively, and transcript abundance of five genes was examined by the qRT-PCR and compared with the expression level from RNA-seq. The primers used were present in Supplementary Table 1. No reverse transcriptase (NRT) and no template control (NTC) served as negative controls.

## Results

### Gut Commensal Bacteria Caused an Acute and Virulent Infection in Hemocoel

**FIGURE 2:**
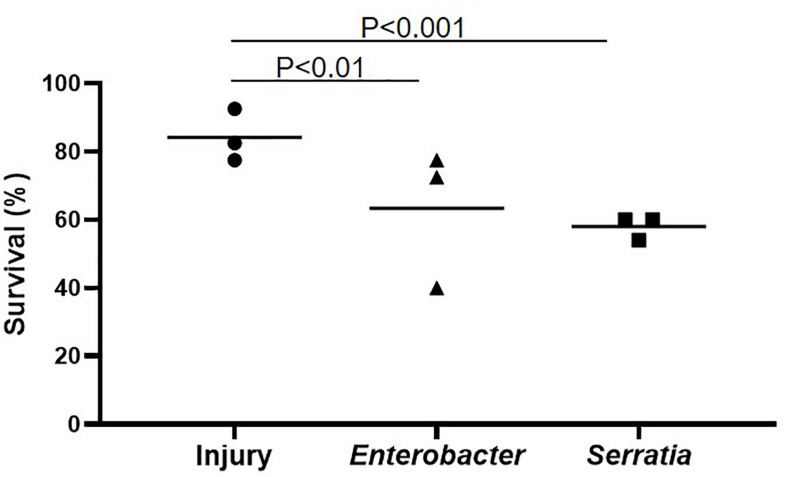
Bacterial infection resulted in mortality. Naive mosquitoes were infected with *Serratia or Enterobacter* by intrathoracical injection. Sterile water injection was used as injury control. The average survival was generated from three replicates. Each replicate had 40 females. The survival was significantly reduced by *Serratia* and *Enterobacter* infection in comparison with injury, tested using Chi square.

The bacterium *Enterobacter* sp. Ag1 was isolated from the midgut of *An. gambiae* in the lab, and *Serratia fonticola* S1 was isolated from the midgut of wild-caught *Aedes albopictus*. Both bacteria are Gram-negative bacteria in order Enterobacterales. *Enterobacter* belongs to family Enterobacteriaceae, and *Serratia* belongs to family Yersiniaceae. The *Enterobacter* is persistently associated with G3 strain in our insectary (Jiang et al., 2012; Pei et al., 2015). We examined the presence of *Serratia* in the gut of the G3 mosquitoes using a PCR assay targeting two *Serratia* genes, *DNA gyrase subunit A*, and *glucose-1-phosphatase*. Metagenomic DNA from the whole body of larva and pupa, the midgut of sugar-fed mosquitoes (day 4 post eclosion) and day 4 post blood-feeding were subject to the PCR assay. Mosquito gene *rpS5* and *Enterobacter* gene *DNA gyrase subunit A* were used as a positive control. The mosquito *rpS5* amplicon was present in all four samples, and the *Enterobacter* gene was not detected in larva and pupa but was present in the adult midgut before and after blood feeding. However, none of the two *Serratia* genes was amplified in the four samples (Supplementary Figure 1). We concluded that the *Serratia* strain was not associated with the G3 mosquitoes in our insectary. Therefore, in this study, the *Enterobacter* strain represents a bacterium that has been associated with the G3 colony, and the *Serratia* strain represents a bacterium that has limited or no association with the G3 colony. Next, we tested the infection outcome of these two bacterial strains in the G3 mosquitoes. Injection of the bacteria, ∼100 CFU per mosquito, into the hemocoel caused an acute hemocoelic infection, almost all infected mosquitoes died in 3 days post injection, therefore survival at 24 h was the most informative data point to present infection outcomes. As shown in Figure 2, *Enterobacter* or *Serratia* injection resulted in an infection with a survival rate of 63.3% and 58.0% at 24 h post injection, respectively, while the injury control (injected with sterile water) had a survival rate of 84.2%. This shows that the two gut symbiotic bacteria can cause an acute virulent hemocoelic infection. *E. coli* as a representative of Gram-negative bacteria in family Enterobacteriaceae has been widely used in the studies of mosquito immunity. *An. gambiae* can tolerate *E. coli* in a large quantity in the hemocoel and survive up to 30 days until all die (Powers et al., 2020), which represents a chronic infection course. Therefore, the infection course and outcome are quite different between *E. coli* and the two gut bacteria, *Enterobacter* and *Serratia.* Therefore, we further studied the mosquito response to the acute and virulent infection model caused by the two bacteria.

### Bacterial Feeding Primed Immunity Against Homogeneous Bacterial Infection

**FIGURE 3:**
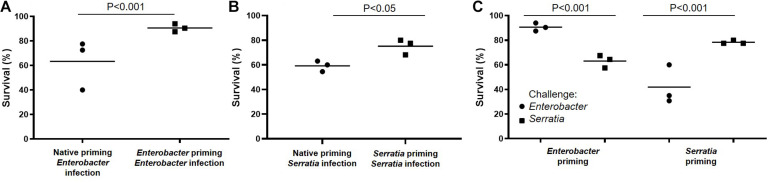
Priming-enhanced antibacterial immunity was specific to the homogeneous bacteria. **(A)** Compared to native microbiota, the *Enterobacter* priming increased mosquito survival upon the *Enterobacter* challenge. **(B)** Compared to native microbiota, the *Serratia* priming increased survival upon the *Serratia* challenge. **(C)** *Enterobacter* priming increased the survival upon the *Enterobacter* challenge but not the *Serratia* challenge. The *Serratia* priming increased the survival upon the *Serratia* challenge but not the *Enterobacter* challenge. The survival data of each challenge were generated from three replicates, each had 40 mosquitoes. The survival difference was compared using Chi square test.

Mosquito midgut harbors a microbiota with various microbes. Previously, we have shown that feeding newly emerged mosquitoes with a sugar diet supplemented with a single bacterium can make the bacteria dominant in the gut microbial community (Pei et al., 2015). We tested the immune priming effect of single bacterial feeding on immunity against bacterial challenges. The scheme of priming and challenge was present in Figure 1. Newly emerged mosquitoes were fed with sugar meal supplemented with *Serratia* or *Enterobacter* (at a concentration of OD_600_ = 1.0) for 3 days. The mosquito cohort with the sugar meal without bacterial supplement was defined as priming with native microbiota. On day 4, the primed mosquitoes were challenged with *Enterobacter* or *Serratia*. Compared to the cohorts with native priming, the *Enterobacter* primed mosquitoes had a better survival upon the *Enterobacter* challenge (90.7 vs. 63.3%, Figure 3A), and the *Serratia* primed mosquitoes had a better survival upon the *Serratia* challenge (77.5 vs. 59.2%, Figure 3B). Then, we examined the specificity of priming effect against challenges using heterogeneous bacteria. The *Enterobacter* primed mosquitoes had a survival of 90.7% upon the *Enterobacter* challenge and 63.1% upon the *Serratia* challenge. Similarly, the *Serratia* primed mosquitoes exhibited a survival of 78.3% upon the *Serratia* challenges and 41.9% upon the *Enterobacter* challenge (Figure 3C). Overall, the bacterial priming via diet enhances antibacterial immunity, the trained immunity demonstrates a stronger protection against challenges with the homogeneous than the heterogeneous bacteria, suggesting that priming is specific at a certain level.

### Transcriptomic Response to Bacterial Priming and Challenge

To identify systemic transcriptomic response to the priming and the bacterial challenge following priming, we conducted RNA-seq to compare transcriptomes in these 10 different conditions (Figure 1): priming without challenge (native microbiota, *Enterobacter*, and *Serratia* priming), priming plus challenge (native, *Enterobacter* and *Serratia* priming plus *Enterobacter* challenge; and native, *Enterobacter* and *Serratia* priming plus *Serratia* challenge) and injury control on mosquitoes with native microbiota.

#### Overview of Transcriptomic Responses to Priming and Bacterial Challenge

**FIGURE 4:**
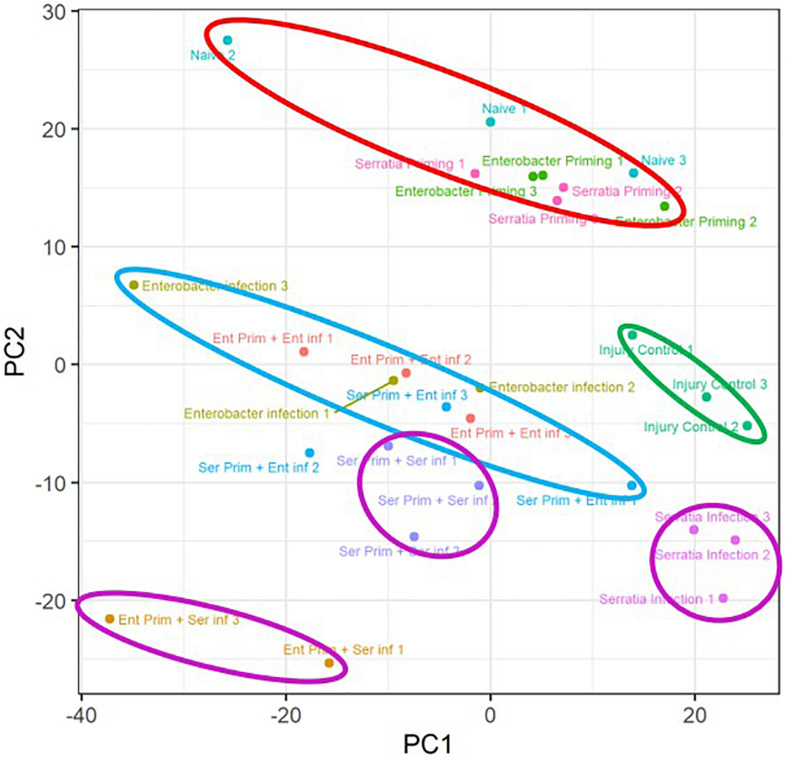
Overview of the priming effects on the transcriptomic response to respective bacterial challenges. Transcriptome replicates from primed cohorts without challenge were marked with a circle in red. Replicates of injury controls were marked with a circle in green. Replicates of *Enterobacter* challenged cohorts with respective priming were marked with a circle in blue. Replicates of *Serratia* challenged cohorts with respective priming were marked with a circle in purple. Naive, cohorts with native microbiota; Enterobacter infection, *Enterobacter* challenged cohort with native microbiota; Serratia infection, *Serratia* challenged with native microbiota.

A principal component analysis was conducted to observe the transcriptomic response to the priming regimes and the impact of priming on transcriptomic response to the bacterial challenge (Figure 4). The transcriptome replicates from the primed cohorts without challenge were clustered closely with a distance from the other conditions (marked by a red circle). Intra-replicate variation of the cohorts with native microbiota appears to be higher than the other two primed cohorts, which may be related to the diverse microbial structure in the native microbiota. The injury controls (marked by a green circle) were separated from the cohorts with the bacterial challenge. The priming effect on the *Serratia* challenge was demonstrated by a clear separation of the three clusters (marked by three purple circles), suggesting the three priming regimes had distinctive effects on the *Serratia* challenge. The replicates of the *Enterobacter* challenge with respective priming regimes (marked by a single light blue circle) were clustered nearby with an interspersed pattern, suggesting that these priming regimes may have overlapping effects on the *Enterobacter* challenge. The transcriptomic patterns were corroborated by qRT-PCR data with five genes in six conditions. The folder changes in RNA-seq data and qRT-PCR were compared, the expression patterns of *DEF1, LYSC1*, and *CLIPA14* were consistent in all six conditions between the two types of data; and *PGRPLB* and *CLIPB12* were consistent in four of six conditions, respectively (Supplementary Figure 2).

#### Transcriptomic Responses to the Priming Without Challenge

**FIGURE 5:**
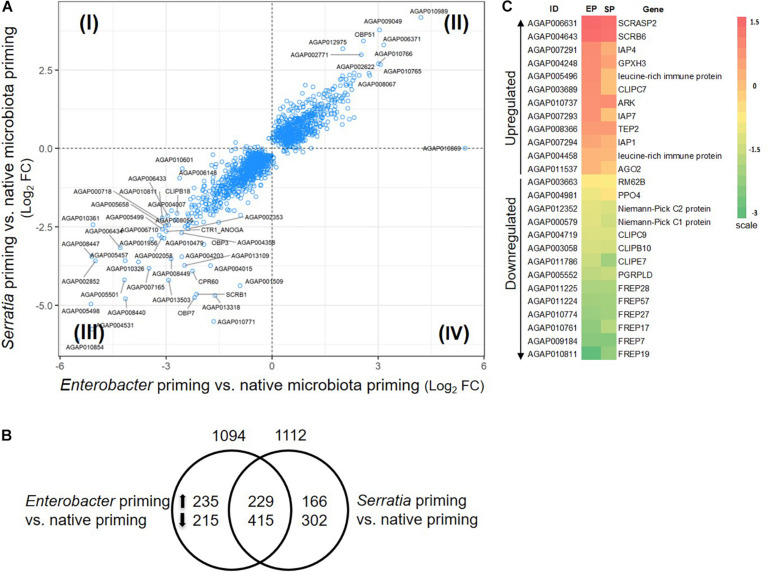
Priming effect of single bacterial diet on the transcriptome without bacterial challenge. **(A)** Transcriptomes in the cohort with *Enterobacter* or *Serratia* priming showed a similar response pattern, majority of genes were regulated in the same direction, plotted in quadrant II and III. Plotted are genes with *q*-value less than 0.05 in either comparison to the cohort with native microbiota. Labeled genes have log_2_ fold change (FC) in either comparison greater than 2.5 or less than –2.5. **(B)** Distribution of the genes that were altered by individual priming. Total number of altered genes is placed on top of the circle, the number of up- or down-regulated genes were specified inside the circle. **(C)** The heatmap illustrates the immune genes that were affected by the *Enterobacter* (EP) and *Serratia* (SP) priming compared to the native priming. The color scale represents the log_2_ fold change between the EP or SP and native community.

The mosquitoes that were fed with regular sugar meals had native microbiota, which was defined as a native primed cohort. The mosquitoes that were fed with sugar meals supplemented with *Enterobacter* or *Serratia* were defined as *Enterobacter* or *Serratia* primed cohort. Compared to the native priming, the *Enterobacter* and *Serratia* priming altered expression of 1094 and 1112 genes, respectively, totaling 1562 genes, among them, 644 genes were affected by both priming regimes (Figures 5A,B). There were 175 immune genes that were affected by either or both priming regimes, accounting for 11.2% of the affected genes. Figure 5C presents a heatmap illustrating 12 upregulated and 14 downregulated immune genes, which were affected by both *Enterobacter* and *Serratia* priming in the same direction. The upregulated genes include the ones that encode three inhibitor of apoptosis proteins (IAPs), two leucine-rich immune proteins, and TEP2, and the downregulated genes include the ones that encode PGRPLD, PPO4, three CLIP serine proteases, two Niemann-Pick proteins, and six FREPs. In the non-immune categories, the upregulated group includes genes encoding seven ATP-binding cassette transporters, three cuticular proteins, 10 cytochrome P450, and 98 unspecified genes, while the downregulated group includes the genes encoding four cuticular proteins, 10 cytochrome P450 proteins, and 135 unspecified genes. The detailed comparison of gene expression in different conditions was provided in Supplementary Table 3 with gene ID and available gene annotation. Overall, the individual priming affected a set of genes that were uniquely responsive to the priming bacteria as well as a set of genes that were responsive to all priming regimes. The affected genes were dispersed in broad categories with diverse functions, many genes were unspecified, no function information was available yet.

#### Infection Responsive Genes in the Cohorts With Native Microbiota

**FIGURE 6:**
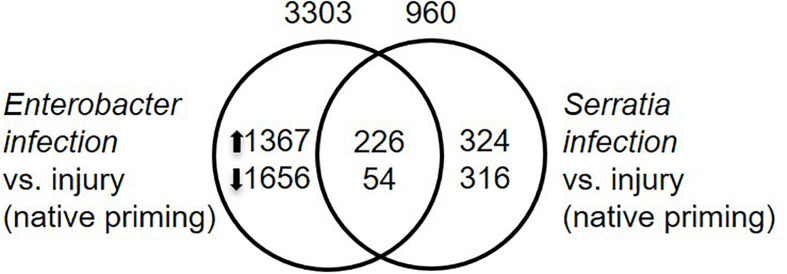
Infection responsive genes. Infection responsive genes were identified by comparison between respective infection vs. injury control with *q*-value < 0.05 as cutoff. Total number of transcriptionally altered genes was specified on top of the circle, number of up- or down-regulated genes were specified inside the circle.

To identify infection responsive genes, we compared transcriptomes between the infected mosquitoes and injury control. The mosquitoes with their native microbiota were used for this purpose. The mosquitoes were challenged with *Enterobacter* or *Serratia*, or sterile water. The *Enterobacter* infection altered the expression of 3303 genes while the *Serratia* infection altered the expression of 960 genes. A set of 320 genes were affected by the two infections commonly, 226 were upregulated and 54 were downregulated by both infections (Figure 6 and Supplementary Table 3). In the immune category, 55 genes were induced by both infections, including the antimicrobial genes, such as *Def1, CecA, GNBPs*, and *lysozyme*, and only two immune genes, *PPO9* and *CLIPB12*, were downregulated, indicating that the infections trigger a typical immune response to the bacteria. The presence of different sets of responsive genes between the two infections suggests that different bacteria can induce different responses, these genes are involved in various processes, which may affect infection outcomes in different ways.

#### Transcriptomic Responses to the Priming Plus Challenge

**FIGURE 7:**
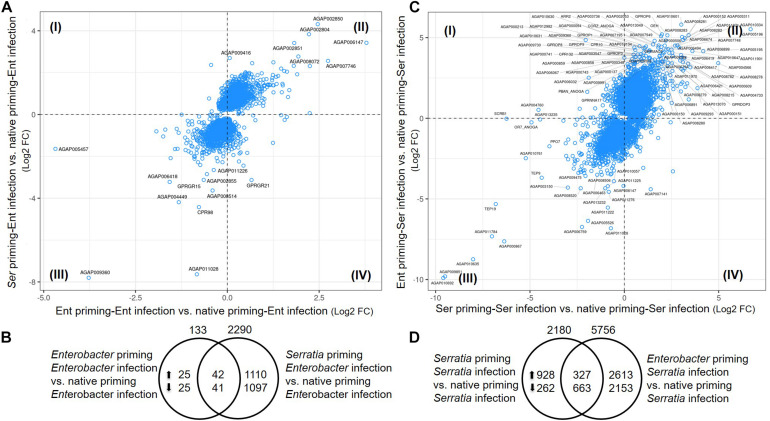
Priming effects on transcriptional response to bacterial challenge. **(A,C)** Comparison between the cohort with *Enterobacter* or *Serratia* priming vs. the cohort with native microbiota. Plotted are genes with *q*-value < 0.05 in either comparison to cohort with native microbiota. Labeled genes have log_2_ fold change in either comparison >2.5 or <–2.5 in **(A)** and >3.5 or <–3.5 in **(C)**. The genes in quadrant I and IV were the genes differentially expressed upon different priming regimes. **(B,D)** Distribution of the genes that were altered by priming. Total number of altered genes is placed on top of the circle, number of up- or down-regulated genes were specified inside the circle. The genes in the central area were shared by both cohorts with respective priming.

**FIGURE 8:**
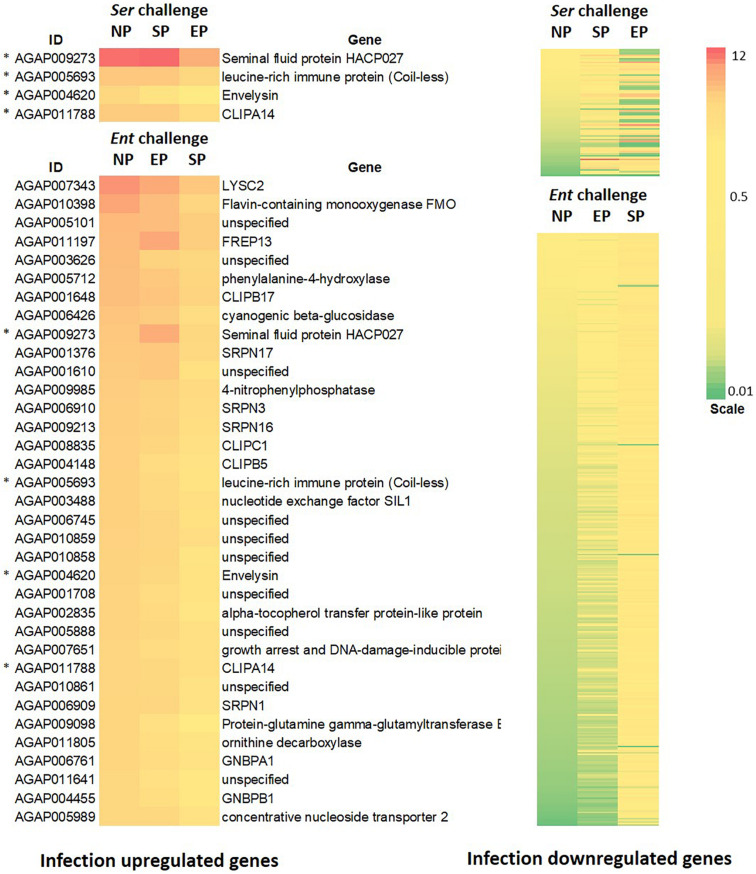
Impact of prior homogeneous and heterogeneous priming on the infection responsive genes. In the *Serratia* challenged cohorts, the infection induced genes (>2-fold) were downregulated by the *Enterobacter* priming, while the infection repressed genes (<2-fold) were either increased or further decreased. In the *Enterobacter* challenged cohorts, the *Enterobacter* infection induced genes were downregulated by the *Serratia* priming, however, most of infection repressed genes were upregulated by the *Serratia* priming. * marks genes that were affected by both challenges. The gene ID and annotation in the downregulated panel were provided in Supplementary Table 5. The heatmaps represent genes with significant difference between the NP and homogeneous and heterogeneous priming (*q*-value < 0.05).

To identify the effects of prior priming on a particular infection, we compared transcriptomes between the cohort with native microbiota and the cohorts with *Enterobacter* or *Serratia* priming followed by the respective bacterial challenge, as depicted in Figure 1. In the case of *Enterobacter* challenges, the cohort with native microbiota and the cohort with *Enterobacter* priming had similar transcriptomic responses, only 133 genes were expressed differentially between, 67 genes were upregulated, and 66 genes were downregulated (Figures 7A,B). On the other hand, the *Serratia* primed mosquitoes resulted in a quite different pattern of responses to the *Enterobacter* challenge, 2290 genes were altered, 1152 genes were expressed with a higher level, and 1138 genes were expressed with a lower level (Figure 7B). In the case of *Serratia* infections, the *Serratia* primed cohort had 2180 genes altered, 1255 genes were upregulated, and 925 genes were downregulated. The *Enterobacter* primed cohort responded to the *Serratia* challenge with 5756 genes altered, 2940 genes were upregulated, and 2816 genes were downregulated (Figures 7C,D). The impact of prior heterogeneous priming on the responsive genes in each challenge was illustrated in Figure 8. In the group of infection-upregulated genes with >2-fold change, the prior *Serratia* priming attenuated the induction of four genes upon the *Enterobacter* challenge, and the prior *Enterobacter* priming reduced the induction level of 35 genes upon the *Serratia* challenge. Interestingly, the four genes were affected by the heterogeneous priming in both challenges, including *CLIPA14* and a leucine-rich immune gene (Figure 8). In the group of infection-downregulated genes with <2-fold change, the *Enterobacter* priming had mixed effects on 71 genes, either increasing or further decreasing the expression level to the *Serratia* infection. The *Serratia* priming affected 377 genes, the expression level of 373 of these genes was increased. Interestingly, only two genes were affected in both scenarios, the expression level of CLIPB12 was further downregulated in both heterogeneously primed cohorts, and the downregulation of AGAP010340, which encodes a Zink finger protein C2H2 type transcription factor, was reversed in both heterogeneously primed cohorts. In the *Enterobacter* repressed gene group, the genes involved in chromosome structure, chromosome transmission, DNA repair, DNA replication, and ubiquitin-proteasome systems were enriched. In the *Serratia* repressed gene group, the genes encoding seven salivary gland proteins were present.

#### Responsive Immune Genes

**FIGURE 9:**
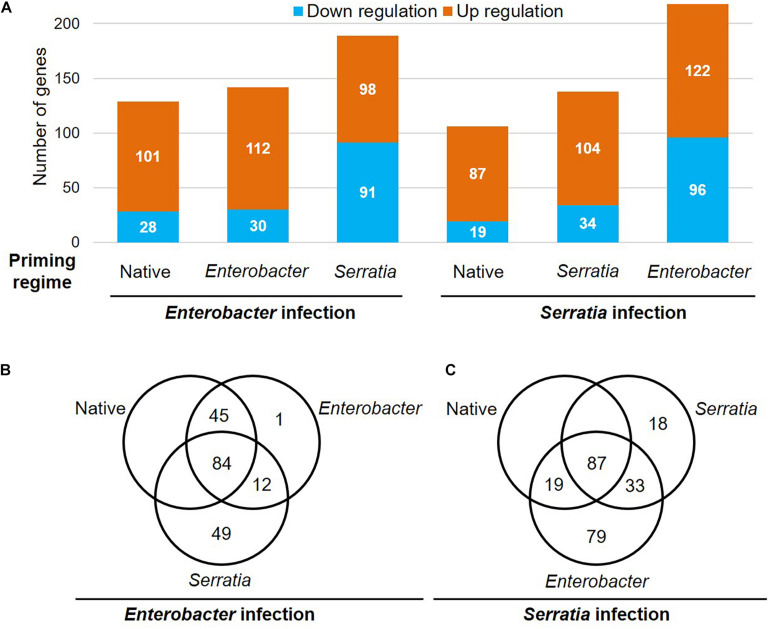
Priming effects on the transcription of the immune genes upon *Enterobacter* and *Serratia* challenge. **(A)** The number of upregulated or downregulated genes is presented in each priming regime upon respective bacterial challenge. **(B,C)** The Venn diagram exhibits genes that were responsive to the respective infection in the cohort with respective priming regime.

To determine the priming effects on immune genes in response to the bacterial challenges, we compared the transcriptional patterns of the immunoDB gene set (Waterhouse et al., 2007). The transcripts of 342 immune genes were detected in our dataset, 279 of them were responsive to the bacterial challenges in at least one of the six cohorts (Figure 9). The gene ID and available annotation were provided in Supplementary Table 4. Compared to the native priming, both *Enterobacter* and *Serratia* priming altered a few dozen more genes upon the challenge with a homogeneous bacterium, however, the challenge with a heterogeneous bacterium resulted in three times more downregulated genes (Figure 9A). Upon the *Enterobacter* challenge, 101 genes were upregulated in the cohorts with native and *Enterobacter* priming. Among these genes, there were 23 CLIP serine protease genes, (seven CLIPA, 10 CLIPB, four CLIPC, and two CLIPE genes), 11 leucine rich immune genes, nine SRPN genes, 13 FREP genes, five GNBP genes (Supplementary Table 4). The *Serratia* priming had a different impact, among the above 101 genes, 36 genes were downregulated upon the *Enterobacter* challenge (Supplementary Table 4). Upon the *Serratia* challenge, in the cohorts with native and *Serratia* priming, 72 genes were upregulated by the *Serratia* challenge, including 21 CLIP genes (17 were shared with *Enterobacter* infection), nine FREP genes, six leucine rich immune genes, eight SRPN genes, and nine TEP genes, and the immune signaling pathway genes *TOLL5A*, *Pelle*, *Cactus*, *IKK2* were upregulated as well. The *Enterobacter* priming resulted in 96 downregulated genes, including 11 genes that were upregulated in the other two primed cohorts (Supplementary Table 4). In all the cohorts 34 genes were upregulated by both *Enterobacter* and *Serratia* challenge, including 13 CLIP genes, five FREP genes, four SRPN genes, and four leucine rich immune genes. These genes were infection inducible, and the priming regimes had little effect on these genes. Interestingly, no downregulated genes were shared by all six cohorts.

In the category of non-immune genes, it worth noting that the genes encoding vitellogenin A1 precursor, vitellogenin and cathepsin B precursor were downregulated by the priming before the challenge, and these genes remained downregulated upon bacterial challenges no matter the priming background (Supplementary Table 4).

## Discussion

Innate immunity in invertebrates can be trained by priming to execute immune defense in an enhanced mode (Milutinovic et al., 2014; Brown and Rodriguez-Lanetty, 2015; Dhinaut et al., 2018; Gourbal et al., 2018; Ferro et al., 2019), which exhibits the high flexibility and plasticity of innate immunity. The innate immune system in invertebrates has demonstrated a widening spectrum of immune memory and specificity, which have led to the reassessment of the definition of immune memory and specificity (Milutinovic and Kurtz, 2016; Gourbal et al., 2018; Melillo et al., 2018; Sharrock and Sun, 2020). *Anopheles* mosquitoes transmit malaria, and antimalarial immunity has been studied extensively. Bacteria-primed immune enhancement against malaria has been well documented (Dong et al., 2009; Rodrigues et al., 2010; Gendrin et al., 2015; Bai et al., 2019; Cappelli et al., 2019). Antibacterial immunity has become a focus in understanding immune priming and underlying mechanisms (Hillyer and Estevez-Lao, 2010; Moreno-Garcia et al., 2015; Barletta et al., 2019; Brown et al., 2019; Powers et al., 2020). Bacterial symbionts are associated with mosquitoes throughout the evolution, the interactions between bacteria and host mosquitoes have been shaping the mosquito immune system. In this study, we explored the priming effect of gut bacteria on the systemic immune response to the hemocoelic infection caused by these bacteria.

The strains of *Enterobacter* and *Serratia* used in this study are gut commensals, which are naturally associated with mosquitoes. However, once being introduced into hemocoel by injection with approximately 100 CFU per mosquito, both strains cause acute virulent infections (Figure 2), and none of the challenged mosquitoes survived beyond 3 days. *Escherichia coli* has been widely used as a representative of Gram-negative bacteria for studying antibacterial immunity in mosquitoes (Moita et al., 2006; Nakhleh et al., 2017; Das De et al., 2018; Brown et al., 2019; Reyes Ruiz et al., 2019; Estevez-Lao et al., 2020; Powers et al., 2020). According to the literature, *E. coli* causes a chronic infection in the hemocoel. In a recent study, *E. coli* infected *An. gambiae* mosquitoes could have a bacterial load up to >300,000 CFUs per mosquito on day 3 and day 7 post bacterial injection (Powers et al., 2020). It appears that the mosquitoes were tolerant to *E. coli*, but not able to clear the infection, the bacteria were persistent in the hemocoel until all mosquitoes died, though about 5% of infected mosquitoes survived exceeding 31 days post infection (Gorman and Paskewitz, 2000; Powers et al., 2020). We observed a similar infection outcome, in which the mosquitoes were injected with 100 nl of *E. coli* in an amount of OD_600_ = 1, and approximately 20% infected mosquitoes survived through 10 days post injection (data not shown). Therefore, the acute infection caused by the two gut commensal bacteria represents an infection model distinct from the one caused by *E. coli.* Regarding taxonomy, *Escherichia* and *Enterobacter* both belong to family Enterobacteriaceae, and *Serratia* belongs to family Yersiniaceae, the two families are in order Enterobacterales. It would be interesting to elucidate what is behind the difference between acute and chronic infections in terms of bacterial virulence factors and mosquito factors in future studies.

Then we used the infection model to examine the priming effect on infection outcomes from the following hemocoelic challenge. The mosquitoes were primed with a sugar meal supplemented with *Enterobacter* or *Serratia* and later challenged with homogeneous or heterogeneous bacteria. Compared to the infection in the cohorts with native gut community, the single bacterium-primed cohorts exhibit increased survival at 24 h post challenge with homogeneous but not heterogeneous bacterial strain used in priming (Figure 3). The data suggest that gut symbionts, when being dominant in the gut community, can train the mosquitoes to enhance immune responses to systemic infection with specificity to a certain degree.

To characterize the transcriptional impact posed by priming, we interrogated the transcriptomes in 10 different conditions, i.e., three priming regimes, six conditions of priming plus challenge with the homogeneous and heterogeneous bacterium, and injury control, as shown in Figure 1. In comparison to native priming, each bacterial priming significantly altered the expression of approximately 1100 genes, and 644 genes were affected by both (Figure 5). These transcriptional changes demonstrate a measurable priming-mediated systemic impact, suggesting that gut microbial shifts via diet manipulation can be sensed and transduced into a systemic response. This finding is corroborated by a recent study, which showed that two different strains of *Serratia*, once orally introduced into *An. stephensi*, could induce different transcriptomic responses to blood meal (Bai et al., 2019). The microbial structure of gut microbiota is diverse and dynamic during the mosquito life cycle (Wang et al., 2011). These dynamic interactions may have connections with various physiological traits. In the genes that are affected by the priming regimes, besides 40 or 50 immune genes, there are >1000 non-immune genes. Cytochrome P450 proteins are largely involved in xenobiotic defense (Feyereisen, 1999). The P450 system operates xenobiotic sensing and defense in the gut, which plays a critical role in maintaining gut homeostasis (Collins and Patterson, 2020). A recent study demonstrates that gut microbes regulate P450 gene expression and affect host pesticide metabolism in the honey bee (*Apis mellifera*) (Wu et al., 2020). In the primed mosquitoes, some P450 genes were upregulated, and some were downregulated, suggesting that the shift of gut microbial composition can be sensed and the P450 machinery is adjusted accordingly. It is worth noting 15 genes encoding solute carrier transporters (Supplementary Table 3). Increasing evidence has emerged that solute carrier transporters play critical roles in nutrient uptake, ion influx/efflux, and waste disposal, which mediates energy and metabolic support for immune activities (Song et al., 2020). Further study is needed to explore the priming effect on immunometabolism, which has become a hot research area recently (Penkov et al., 2019; Samaddar et al., 2020).

To identify the priming effect on transcriptomic responses to the bacterial challenge, we compared the transcriptomes of cohorts with three different priming regimes: native microbiota, *Enterobacter*, and *Serratia*. In the mosquitoes with native microbiota, the *Enterobacter* challenge altered more genes than *Serratia* did (Figure 6). In the priming contexts, the *Enterobacter* primed cohorts had a similar response as the native primed cohort did, only 133 more genes were differentially affected. However, the *Serratia* priming affected much more genes than the native priming did (Figures 7A,B). In the case of *Serratia* challenge, the *Serratia* priming altered more genes than the native priming, and the *Enterobacter* priming had a much broader influence than the other two priming regimes did (Figures 7C,D). It appears that the primed mosquitoes responded more drastically to a heterogeneous bacterial challenge than to a homogeneous challenge. A transcriptomic response involving more genes may reflect chaotic dynamics, which may not necessarily result in a beneficial outcome. Indeed, phenotypically, the primed immune protection is associated only with homogeneous challenges (Figures 2, 3). In the immune gene category, many immune genes are responsive to the challenges (Figure 9 and Supplementary Table 4), including genes encoding microbial pattern recognition, immune signaling, antimicrobial peptides, FREPs, CTLs, PPOs, CLIP serine proteases, and Serpins. Many of these genes play different roles in modulating melanization, one of the defense mechanisms (Christensen et al., 2005; Dong and Dimopoulos, 2009; An et al., 2011; Cao et al., 2017; Gendrin et al., 2017; Meekins et al., 2017; Kumar et al., 2018). The PPOs are activated by proteolytic cleavage via an enzymatic cascade of serine proteases. This process requires complex interactions of different members in the CLIPs B, C, and A as well as serpins (An et al., 2011; Gulley et al., 2013; Povelones et al., 2013; Zhang et al., 2015; Cao et al., 2017; He et al., 2017; Meekins et al., 2017; Nakhleh et al., 2017; El Moussawi et al., 2019; Sousa et al., 2020). It has been shown recently that microbial melanization can be triggered by *E. coli* infection (Sousa et al., 2020). The genes that participate in modulating melanization were enriched in the transcriptomes responsive to the priming regimes. It would be interesting to further investigate priming effects on the modulation of melanization in response to bacterial infections.

Besides typical immune genes, we noticed a set of genes with annotated functions related to lysosomes. In addition to six genes encoding lysozyme C, two genes encoding Niemann Pick type C1 (NPC1) and nine genes encoding Niemann Pick type C2 (NPC2) were responsive to the bacterial challenges in different priming regimes (Supplementary Table 4), and genes encoding cystinosin and mosGILT were responsive to the challenges as well. Cystinosin is a cystine/H(+) symporter that exports cystine out of the lysosomes and is involved in melanin synthesis (Kalatzis et al., 2001; Chiaverini et al., 2012). The mosGILT, INF-γ inducible lysosomal thiol reductase, has been shown to play a critical role in ovarian development. The mosaic mosGILT-mutant mosquitoes exhibit an impaired 20E secretion in the ovaries and downstream vitellogenin synthesis in the fat body (Yang et al., 2020). The reduction of Vg protein, in turn, favors TEP1 mediated *Plasmodium* killing since the Vg interferes with TEP1 binding to ookinetes (Rono et al., 2010). Interestingly, the *Vg* expression is repressed by Rel1 and Rel2 (Rono et al., 2010). In the current study, the *Vg* expression was downregulated by both *Enterobacter* and *Serratia* priming as well as bacterial challenges (Supplementary Table 4). In honeybee *Apis mellifera*, Vg plays a dual role in reproduction and immunity. The Vg has immunological binding properties, it can bind to both Gram-positive bacterium *Paenibacillus larvae* and Gram*-*negative bacterium *E. coli*, and microbial pattern molecules lipopolysaccharide and peptidoglycan as well. More interestingly, pieces of *E. coli* cell wall can be carried into developing eggs by the Vg, which demonstrated the participation of Vg in the *trans-*generational immune priming in the honeybee (Salmela et al., 2015). It would be interesting to investigate the roles of Vg mediated immunity in mosquitoes in different conditions, for example, before, during, and after blood feeding. The Niemann Pick type C1 (NPC1) is an integral transmembrane protein of the limiting membrane of the lysosome. The Niemann Pick type C2 (NPC2) is a soluble cholesterol binding protein. In humans, the mutation of NPC genes can cause lysosomal storage diseases, which result in inflammation and altered innate immune response (Platt et al., 2016; Rigante et al., 2017). Lysosomes process various substrates from phagocytosis, endocytosis, and autophagy. Mosquito Vg is processed by vitellogenic cathepsin B, a lysosomal thiol (cysteine) protease (Cho et al., 1999; Moura et al., 2015). In short, our transcriptome data imply the connections of multiple lysosomal genes to the immunity in mosquitoes.

In this study, we show that the mosquito antibacterial immunity can be enhanced by priming using gut bacterial symbionts via sugar meals. The priming-trained immunity demonstrates certain specificity. The priming effects systemic transcriptomic responses to the following challenges. When primed mosquitoes were challenged by a heterogeneous bacterium, more complex transcriptomic responses occurred, but no phenotypic protection was observed. In addition to typical immune genes, many non-immune genes are affected as well, suggesting that the priming effects are diverse and systemic. Hemocytes are key immune players. In this study, the whole mosquito transcriptomes were profiled, which largely access the transcriptomes in fat body cells and other cells that were sufficiently represented in the samples. Unfortunately, such RNA-seq data do not have the resolution to tease out responses of hemocytes in the context. There are cross-talks between midgut, fat body, and hemocytes during an immune response (Das De et al., 2018). The genes identified in the current study would be the targets of future studies to elucidate the mechanisms behind the priming effects. The infection outcome of the acute hemocoelic infection caused by the two gut commensal bacteria is different from the outcome of the chronic infection caused by *E. coli.* This warrants further studies to elucidate what is behind the differences. Lastly, we would like to emphasize that the *Enterobacter* strain is associated with G3 strain in our insectary, while the *Serratia* strain was derived from wild *Aedes* mosquitoes. And the *Serratia* strain was not detected in the G3 colony (Supplementary Figure 1), suggesting that the strain is not associated with the G3 mosquitoes in our insectary. Therefore, the differences in the infection pattern and transcriptomic response between the two bacterial strains may also attributed to the fact that the *Serratia* strain is not a regular gut resident in the G3 mosquitoes. In summary, this study presented novel data that furthered the understanding of mosquito immunity.

## Data Availability Statement

The datasets presented in this study can be found in online repositories. The names of the repository/repositories and accession number(s) can be found below: NCBI SRA (accession: PRJNA691571).

## Ethics Statement

The animal protocol was reviewed and approved by the NMSU IACUC.

## Author Contributions

JX conceived the study and wrote the manuscript. JX and AK designed the experiments. AK, AP, WY, JC, AM, and JX conducted the experiments and data analysis. PT, SC, and JX analyzed the transcriptome data. All authors contributed to the article and approved the submitted version.

## Conflict of Interest

The authors declare that the research was conducted in the absence of any commercial or financial relationships that could be construed as a potential conflict of interest.

## Supplementary Material

Supplementary Figure 1 Bacterial detection in the gut. **(A)** The gut metagenomic DNA was extracted from 3rd to 4th instar larvae, pupae, 5-day old female adults, and 4 days post blood meal. The presence of *Serratia* and *Enterobacter* was examined by PCR targeting two *Serratia* genes (DNA gyrase and glucose phosphatase) and one *Enterobacter* gene (DNA gyrase). Mosquito gene rpS5 PCR was positive for all stages. *Enterobacter* was detected in adult gut only, *Serratia* was not detected in any stages. **(B)** The specificity of bacterial amplicons was determined by respective bacterial genomic DNA. The PCR was positive with respective bacterial DNA. No non-specific amplifications occur.

Supplementary Figure 2 The expression patterns of five genes were compared between RNA-seq and qPCR. The data were presented as fold change. In the *Enterobacter* challenged mosquitoes, fold changes of (EP-EI)/(NP-EI) and (SP-EI)/(NP-EI) were presented. In the *Serratia* challenged mosquitoes, fold changes of (SP-SI)/(NP-SI) and (EP-SI)/(NP-SI) were presented. In most of the six conditions, the gene expression patterns were comparable between RNA-seq and qPCR. The *PGRPLB* was not consistent in two conditions, and *CLIPB12* was not consistent in two conditions, these inconsistent conditions were marked by arrows. Error bar represents standard deviation.
