# Supplementary figures and images for: Trained Immunity in Anopheles gambiae: Antibacterial Immunity Is Enhanced by Priming via Sugar Meal Supplemented With a Single Gut Symbiotic Bacterial Strain
Source: Front Microbiol. 2021 Apr 30;12:649213. doi: 10.3389/fmicb.2021.649213 (PMC8121176; doi:10.3389/fmicb.2021.649213)

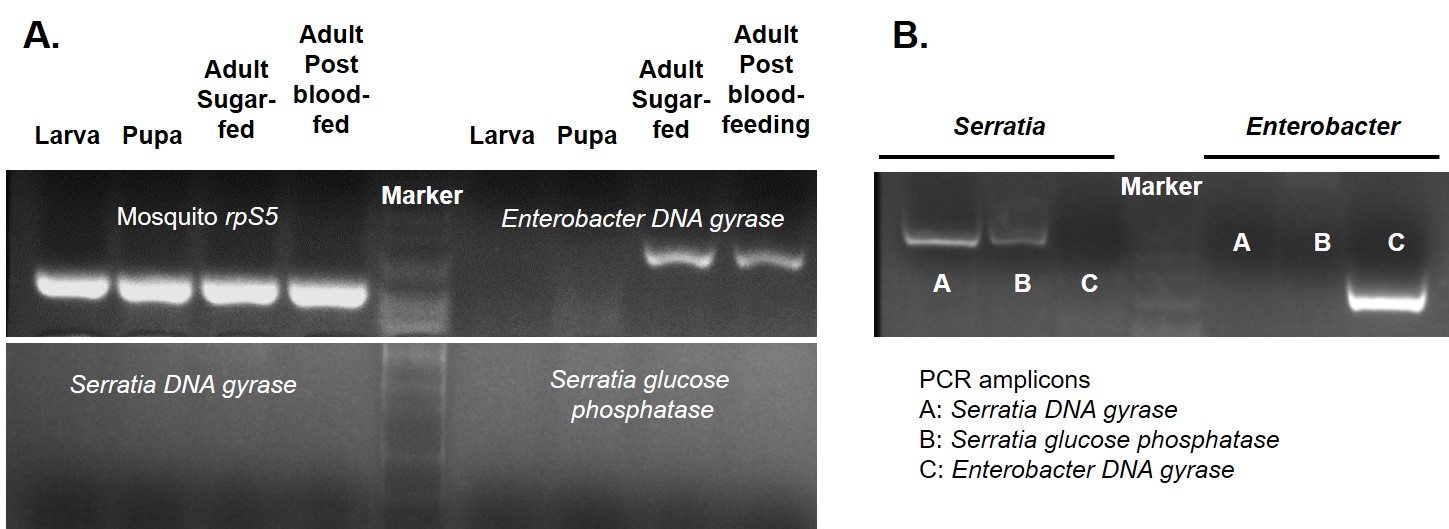

Supplement: Supplementary Figure 1 — Bacterial detection in the gut. (A) The gut metagenomic DNA was extracted from 3rd to 4th instar larvae, pupae, 5-day old female adults, and 4 days post blood meal. The presence of Serratia and Enterobacter was examined by PCR targeting two Serratia genes (DNA gyrase and glucose phosphatase) and one Enterobacter gene (DNA gyrase). Mosquito gene rpS5 PCR was positive for all stages. Enterobacter was detected in adult gut only, Serratia was not detected in any stages. (B) The specificity of bacterial amplicons was determined by respective bacterial genomic DNA. The PCR was positive with respective bacterial DNA. No non-specific amplifications occur. [file Image_1.JPEG]

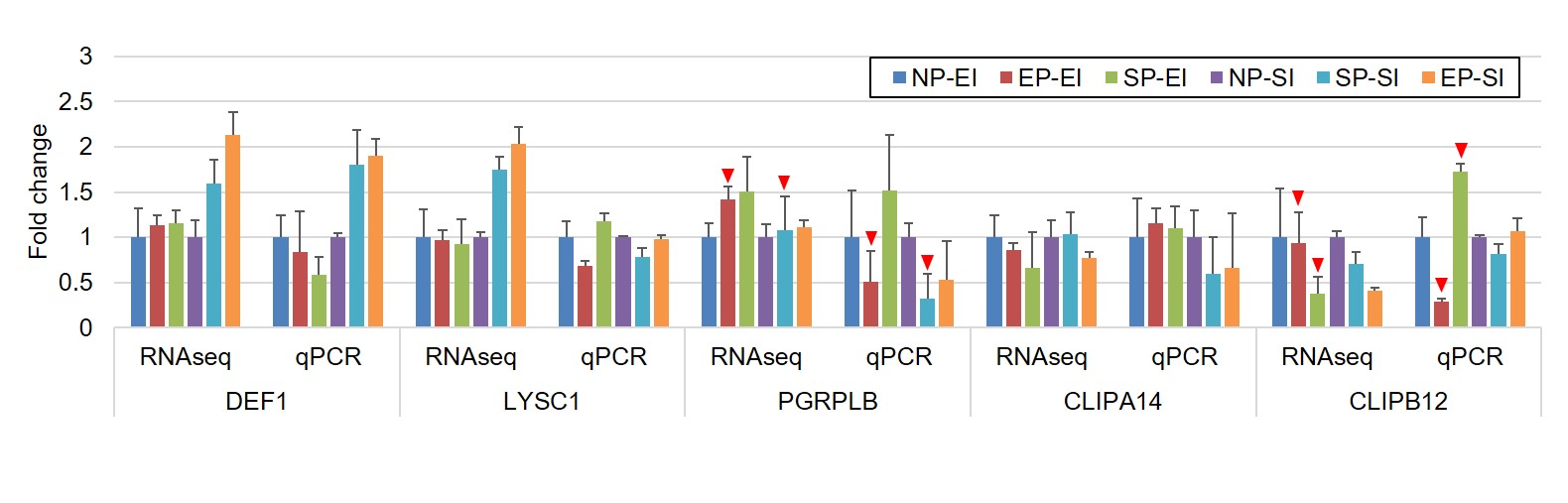

Supplement: Supplementary Figure 2 — The expression patterns of five genes were compared between RNA-seq and qPCR. The data were presented as fold change. In the Enterobacter challenged mosquitoes, fold changes of (EP-EI)/(NP-EI) and (SP-EI)/(NP-EI) were presented. In the Serratia challenged mosquitoes, fold changes of (SP-SI)/(NP-SI) and (EP-SI)/(NP-SI) were presented. In most of the six conditions, the gene expression patterns were comparable between RNA-seq and qPCR. The PGRPLB was not consistent in two conditions, and CLIPB12 was not consistent in two conditions, these inconsistent conditions were marked by arrows. Error bar represents standard deviation. [file Image_2.jpg]
